# A coaching approach to strengthen farm management teams to reduce antimicrobial use in Dutch high usage pig farms: a 2 year intervention study

**DOI:** 10.3389/fvets.2024.1422756

**Published:** 2024-07-29

**Authors:** Heleen Prinsen, Huifang Deng, Dick Heederik, Jaap A. Wagenaar, David C. Speksnijder, Wietske Dohmen

**Affiliations:** ^1^Division of Infectious Diseases and Immunology, Faculty of Veterinary Medicine, Utrecht University, Utrecht, Netherlands; ^2^Institute for Risk Assessment Sciences, Utrecht University, Utrecht, Netherlands

**Keywords:** coaching, antimicrobial use, intervention, biosecurity, vaccination, pig farms, management team

## Abstract

The use of antimicrobials in the pig sector in the Netherlands has been reduced by more than 70% over the last decade. However, there is still a considerable number of pig farms that have not been able to lower their antimicrobial usage (AMU) to a sufficiently low level, comparable to the majority of the other pig farms. Therefore, an intervention study was initiated to lower on-farm antimicrobial use in which 45 pig farms with high AMU were recruited. These farms were coached over a period of 2 years whereby different management interventions were introduced. During the 2-year study period a significant reduction of 13 and 17% in total AMU was seen in weaned piglets and fattening pigs respectively. The introduction of coaching as well as multiple management interventions were (univariably) associated with the decrease in AMU. After mutual adjustment of coaching and individual interventions, the association between coaching and AMU became substantially weaker, indicating that coaching and interventions were interrelated and specific interventions explained the reduction in AMU. In conclusion, a coaching effect was observed in this study, with an effect on AMU through specific interventions. More insights are needed regarding the role and effects of coaching on the influence on the management team comprising the farmer, veterinarian and (feed) advisor, and interventions implemented.

## 1 Introduction

The overuse and misuse of antimicrobials and the subsequent development of antimicrobial resistance (AMR) is a worldwide problem. AMR is considered by the World Health Organization (WHO) and the World Organization for Animal Health (WOAH) as one of the biggest threats to global health, food security and development (1, 2). Antimicrobial use in farm animals has been criticized for contributing to the emergence of resistant bacteria and the spillover of AMR to humans, either through the food chain, direct contact with animals or via the environment (3–5). In Europe, the use of antimicrobials in livestock production still varies considerably between countries (6). Since 2006, Europe banned the use of antimicrobial growth promotors in animals, followed by new regulations in 2019 (with an update in 2022) on increasing transparency to its use, as well as banning routine and preventive use of antimicrobials.

From 2007 onwards, the Netherlands implemented the “Dutch Success Model”; the obligation for farmers to yearly evaluate and write an Animal Health Plan and Animal Treatment Plan together with their veterinarian and the introduction of a one-on-one relationship with a veterinarian with the goal to reduce AMU in farm animals (7). In the meanwhile, an independent Veterinary Medicines Institute (SDa), which monitors and reports AMU in Dutch animal husbandries has been founded. Moreover, a series of other events was initiated, partially by the livestock industry themselves, e.g., a ban on preventive use of antimicrobials, restricting the use of critically important antimicrobials (fluoroquinolones, 3rd- and 4th-generation cephalosporins and colistin). Due to a combination of these compulsory and voluntary actions, AMU was decreased 71.9% in the pig sector in 2022 compared to the reference year of 2009 in the Netherlands (8). However, despite all efforts of the livestock sectors, there is still a considerable number of pig farms with higher than average AMU patterns. For instance, structural high AMU (defined as a usage over a period of at least 2 years above set benchmark values) is seen among 15%−20% of the pig farms (9). The benchmark values are values set by the Dutch Veterinary Medicines Institute to indicate the cut-off value between an acceptable level of AMU vs. a level that requires action to lower AMU.

In recent years, several studies have focused on the effect of management interventions on AMU on farms. For instance, improving biosecurity has been shown to influence the reduction in AMU at the herd level on German farms (10). In a Belgian/Dutch study, improved biosecurity including implementation of different management, housing and feeding measures led to a reduction of AMU in pig farms (11). A risk factor study showed that the level of internal biosecurity and microclimatic conditions in pig farms were associated with AMU (12). In contrast to above-mentioned studies focused on farm practices, there is a growing number of other studies focussed on the behavior of the farmers, risk perception and the influence on AMU (13, 14). Most studies explored attitudes and behavior of the farmers by using questionnaires and in-depth interviews (15–19). In a study done by Speksnijder et al. (20), veterinarians were trained on their communication and advisory skills, and a professional facilitator was added to guide the process of improving farm management and AMU reduction in dairy farms. Houben (21) tested a specific behavior change model in an antimicrobial stewardship study, where veterinary coaches identified farmer specific key elements to change the behavior of farmers to assess the effectiveness of the model. However, one limitation of those type of intervention studies is that the actual action driving the reduction of AMU was not clearly specified. It is clear that factors related to farm management practices and farmers' behaviors both play a role on AMU, research of a combination of these two type of interventions combined to evaluate their effect on AMU is generally not in place, because it involves different study designs, is time consuming and is difficult to estimate the interaction effect.

In this longitudinal study the effect of a specific way of coaching on the use of antimicrobials in high AMU pig farms was investigated. In addition, associations between AMU, coaching and introduced management interventions during the coaching period were explored.

## 2 Materials and methods

### 2.1 Farm selection

The study was conducted between March 2019 and December 2021 on conventional pig farms in the Netherlands. All types of pig farms (sows, weaned piglets, fattening pigs and combinations) were included in the recruitment, but the main focus was on weaned piglets and fattening pigs because of their high AMU. Farms were eligible when AMU was above the national benchmark values of AMU for the pig sector [above 5 DDDA/Y (defined daily dose animal/year) for fattening pigs and above 20 DDDA/Y for weaned piglets, as established by the Dutch Veterinary Medicine Institute (22)]. The farmers together with their veterinarian and feed advisor (entitled the management team) participated. In some of the farm management teams, family members of the farmer, a farm manager and/or farm employees were included, based on their role and participation in the management team. The farms were recruited in several ways by: (a) publishing articles in professional farmers magazines and newsletters; (b) a personal letter to all high antimicrobial users known by the quality system of the sector that monitors the antimicrobial usage, in which farmers were informed about the project and received an update on their farm specific AMU; (c) general meetings with stakeholders in the pig industry; (d) by visiting the five largest veterinary practices with mainly pig veterinarians to motivate them to participate; and (e) by contacting 250 pig farmer members of a farmers organization [Land-en Tuinbouw Organisatie (LTO) Nederland] randomly by phone.

### 2.2 Study design

The study was set up as a stepped-wedge intervention study, which has been used increasingly to evaluate service delivery type of interventions. It involves random and sequential crossover of clusters from control to intervention until all clusters are exposed in different points in time (23). The design includes an initial period in which no clusters are exposed to the intervention. AMU data collection continues throughout the study period, so that each cluster is included as control as well as intervention farm. A total of 45 farms, in five clusters of four to 13 farms were enrolled in the study during a period of 2 years. The intervention period on the farms comprised 15–24 months. The time schedule of the stepped-wedge design is described in Table 1.

**Table 1:** Time schedule of the applied stepped-wedge design for the participating farms, with indication of start and end of the intervention and the number of farms in the different groups.

	**2018**	**2019**				**2020**				**2021**			
		**Jan–Mar**	**Apr–June**	**July–Sept**	**Oct.–Dec**	**Jan–Mar**	**Apr–June**	**July–Sept**	**Oct.–Dec**	**Jan–Mar**	**Apr–June**	**July–Sept**	**Oct.–Dec**
Farm cluster 1	^***^		^*^13										
Farm cluster 2	^***^			^*^9									
Farm cluster 3	^***^					^*^4							
Farm cluster 4	^***^							^*^8					
Farm cluster 5	^***^								^*^11				

^***^Retrieving AM prescription data retrospectively from the quality systems of the sector.

^*^Start of the intervention and the number of participating farms per group.

#### 2.2.1 Supervising management teams and coaching approach

Farm management teams were coached and supervised in their process and collaboration toward less antimicrobial use by an independent coach. Three independent coaches, experienced in coaching and working for LTO Nederland were randomly assigned to the farms and visited the farms 4–6 times during the study period. The coaches were instructed before the start of the study and were guided during the 2 year intervention study by the project coordinator in six-weekly meetings. A systematically designed intervention was developed by using the Intervention Mapping (IM) framework (24). In short, the IM framework was used to identify, analyse and discuss the problem and factors that can influence certain behavior of the farmer or the management team that enhances high antimicrobial use. Subsequently, the behavioral outcomes, behavior of the farmer and management team, that were expected to lower the AMU were defined. In the next step theoretical methods were identified and translated into practical applications which were tailored for the farmers and converted into a toolbox for the coaches. Based on these outcomes, the management team made a plan of action, under supervision of the coach. During each meeting, mostly physical and some online, actions agreed on in earlier meeting were evaluated and discussed under the supervision of the coach.

### 2.3 Data collection

#### 2.3.1 AMU

In the Netherlands, so called national “assigned databases” have been established to register and track all antimicrobial drug deliveries to each farm since 2012. Veterinarians have to register delivered antimicrobials to all livestock farms. For this study, AMU data at farm level and number of animals per age category on the farm were derived from two existing databases for the pig sector (IKB varken and IKB Nederland) from the beginning of 2018 until the end of 2021. Antimicrobial use was expressed as DDDA per farm. The DDDA was used to determine the AMU per farm and per animal category per 100 days. The method to calculate DDDA is explained and discussed in detail in earlier publications (9, 25). In summary, DDDA/100 days was calculated by dividing the total number of treated kilograms on a farm for the relevant observation period (i.e., 100 days in this study) by the average number of kilograms of animals present on that farm for each animal category (i.e., weaned piglets and fattening pigs).

#### 2.3.2 Recording of the intervention data

During the coaching period the farmers carried out the interventions agreed upon by the management team. At every farm visit, relevant data on interventions introduced was collected using a format specifically designed for this project and noting the starting date and the responsible person. In a subsequent meeting it was verified whether implementation had taken place or not. If the agreed intervention was not implemented, data was not included in the analyses. This resulted in a list of 80 different interventions, some partly overlapping, added from all participating farms. These interventions were divided into 13 groups (Supplementary Table 1), based on the literature and consensus during a meeting with veterinary experts, and subsequently used in the data analysis.

### 2.4 Statistical analysis

Linear mixed effects models were fitted to the data to analyse the effect of coaching on the reduction of AMU. For the coaching model, log_10_-transformed DDDAs per 100 days was used as the dependent variable, coaching (yes/no), time period, number of coaching visits, herd size (per thousand), coaches and animal category (weaned piglets or fattening pigs) were used as independent variables in the mixed model. Given the overall decreasing trend in AMU in the Netherlands during the study period (9, 22, 26, 27), the effect of time on changes in AMU on intervention farms was checked specifically. In order to take differences between farms into account, a farm-specific intercept was included as random effect. A first order auto-regressive (AR1) correlation structure was used to take into account correlations between repeated observations within a farm over time. Weights were included into the model to compensate for heterogeneity of variance. If no antimicrobial consumption occurred during a certain period, a small number was assigned (two-thirds of the lowest consumption for the farm involved). Associations between dependent and independent variables were evaluated by univariable analysis. All independent variables with a *p*-value ≤ 0.2 were selected and entered into a multivariable model. The final model was built in a backward elimination process. All variables with a *p*-value < 0.05 were kept in the model. Multicollinearity among explanatory variables was checked using the variance inflator factor (VIF), and variables were excluded if the VIF score was larger than 5. Initially, potential confounding was analyzed univariately. Subsequently, all confounders were analyzed together in a model to evaluate interdependency and select a relevant set of confounders. Residuals of the fitted model were visually checked for normality and conditional *R*^2^ coefficient (proportion of variance explained by both fixed and random effects) was checked for the goodness of fit. A similar structure of the mixed model was used to estimate the effect of different intervention groups measures on the reduction of AMU, with different interventions groups (yes/no) as independent variables. In addition, bivariable linear mixed effects models were used to explore the mutually adjusted effect of coaching and individual intervention measures. Given the overall decreasing trend in AMU in the Netherlands during the study period (9, 22, 26, 27), the effect of time was checked specifically. All models were implemented in R version 4.1.1 (28).

## 3 Results

### 3.1 Farm characteristics

In total, 57 out of ~800 farms with high AMU, agreed voluntarily to participate. A total of 45 farms completed the whole study and were included in the analyses. Twelve farms were excluded from the analyses because they dropped out of the project, stopped farming and/or because of incomplete data. The average number of weaned piglets and fattening pigs on the participating farms during the study period were 3,301 (range 1,050–9,000) and 3,994 (range 800–9,753) respectively.

### 3.2 AMU characteristics

**Figure 1:**
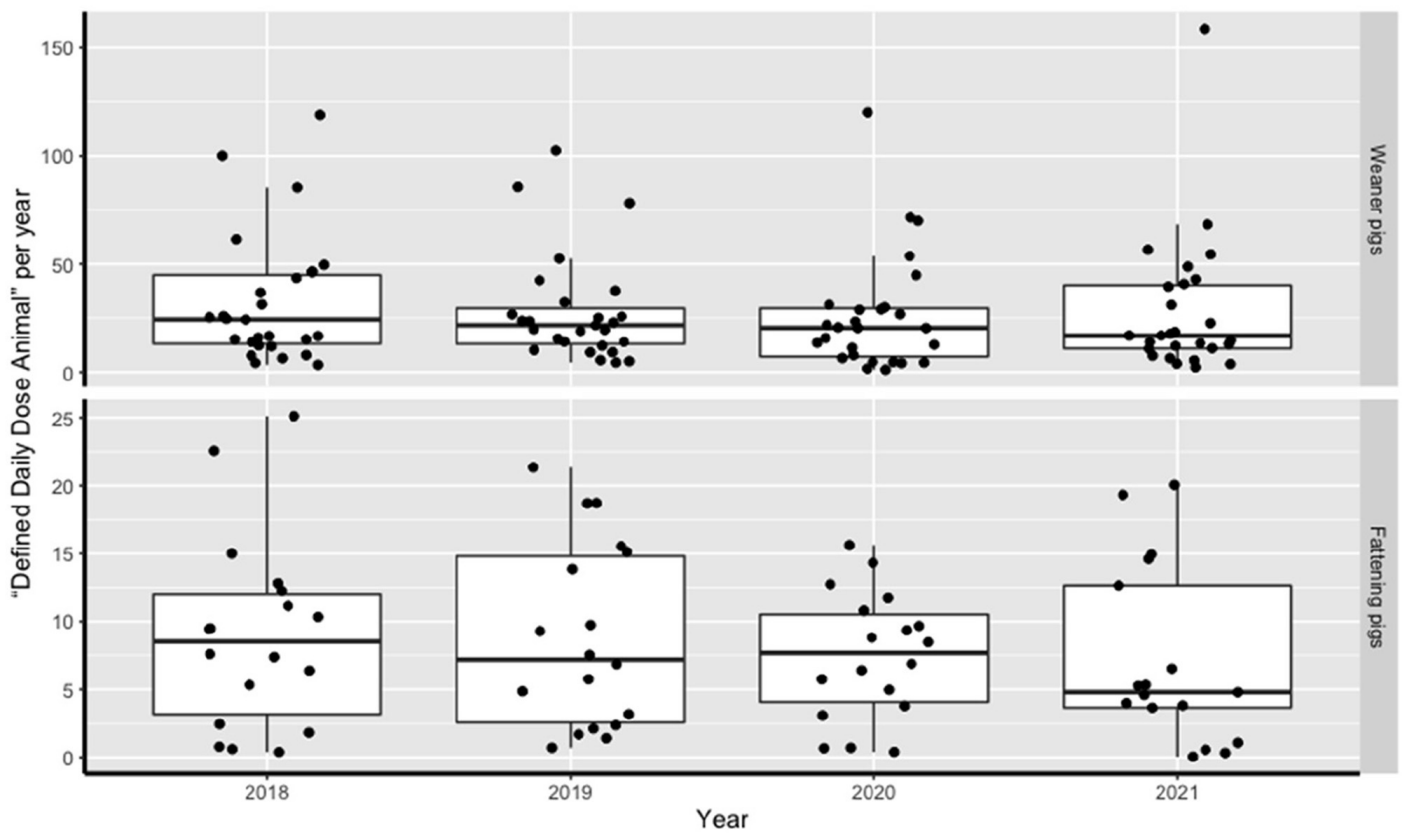
Total DDDA/year at farm level of the participating farms.

Mean AMU between January 2018 and December 2021 in weaned piglets and fatteners was 28.5 (±28.1 SD) and 8.11 (±6.24 SD) DDDA/year respectively. These AMU levels were clearly above the national benchmark levels for these animal categories (DDDA/year of 20 and 5 respectively). A clear decrease in average level of AMU over the 4 years was observed (Figure 1). For weaned piglets, the average use of 32.2 (±29.6 SD) DDDA/year at the start of the study declined to an average of 27.9 (± 31.9 SD) DDDA/year at the end of the study (−13%). For fatteners, an average of 8.8 (±7.0 SD) DDDA/year was observed at the start and 7.3 (±6.4 SD) DDDA/year at the end of the study (−17%).

### 3.3 The effect of coaching on AMU

Coaching was negatively and significantly associated with AMU change during the intervention period expressed as DDDA/100 days in the univariable analysis (−0.07, 95% CI = −0.14 to 0.00). AMU differed between animal categories and was positively associated with herd size. To evaluate the dependency between the effect of coaching and the effect of time, a bivariable analysis was conducted in which coaching and period were mutually adjusted for. In this analysis, the effect of coaching did not change, while the effect of time disappeared, which indicates that coaching had a separate effect on change in AMU. Results from the univariable and multivariable analysis are shown in Table 2. AMU, expressed as DDDA per 100 days was around one unit higher in periods without coaching compared to periods with coaching. Separate analyses for weaned piglets and fattening pigs showed similar effects for coaching (results not shown). There were no significant differences in coaching effect between coaches, coaching visits, and length of coaching.

**Table 2:** Univariable and multivariable analysis for variables associated with AMU in Dutch pig farms.

		**Univariable analysis**			**Multivariable analysis**		
**Variable name**		**Estimated coefficient (EC)**	**Confidence interval (CI)**	***p*** **-value**	**EC**	**CI**	***p*** **-value**
Period		−0.01	−0.01 to 0.00	0.119			
Coaching	Yes	−0.07	−0.14 to 0.00	**0.037**	−0.07^a^	−0.13 to 0.00	**0.042**
	No			Reference			
Herd size^b^		0.07	0.00 to 0.14	**0.035**			

^a^Estimate was adjusted for animal category.

^b^Herd size was rescaled (herd size/1,000). Bold values indicate significant *p*-values.

### 3.4 Effects of interventions on AMU

Univariable analyses using the linear mixed effect model showed that change in feed, adjusting vaccination strategy, treatment, contact structure, internal biosecurity, and development of management team were associated with AMU with a *p*-value < 0.2. These variables were therefore selected and entered in a multivariable model. Animal category was added in the multivariable analysis step. Only two interventions, i.e., adjusting vaccination strategy and improvement of internal biosecurity were statistically significant in the multivariable analysis (Table 3).

**Table 3:** Estimated parameters for the effect of intervention on AMU reduction in Dutch pig farms from the univariable and multivariable analysis.

	**Univariable analysis**			**Multivariable analysis**		
	**Log (DDDA/100 days)**					
**Predictors** ^*^	**Estimates**	**95% CI**	***p*** **-value**	**Estimates**	**95% CI**	***p-*** **value**
Making checklist protocol (yes/no)	−0.03	−0.15 to 0.09	0.604	–	–	–
Biosecurity check (yes/no)	−0.01	−0.34 to 0.31	0.933	–	–	–
Change in feed (yes/no)	−0.11	−0.22 to −0.01	**0.030**	–	–	–
Monitor animal data (yes/no)	0.08	−0.06 to 0.23	0.262	–	–	–
Cleaning (yes/no)	−0.04	−0.22 to 0.14	0.670	–	–	–
Adjusting vaccination strategy (yes/no)	−0.14	−0.23 to −0.05	**0.003**	−0.10	−0.19 to −0.01	**0.027**
Treatment (yes/no)	−0.11	−0.25 to 0.03	**0.116**	–	–	–
Contact structure (yes/no)	−0.10	−0.23 to 0.03	**0.140**	–	–	–
Biosecurity external (yes/no)	−0.12	−0.38 to 0.15	0.384	–	–	–
Biosecurity internal (yes/no)	−0.36	−0.60 to −0.11	**0.005**	−0.27	−0.49 to −0.01	**0.058**
Climate (yes/no)	−0.00	−0.16 to 0.15	0.966	–	–	–
Developing management team (yes/no)	−0.10	−0.21 to 0.01	**0.075**	–	–	–
Change management team (yes/no)	−0.04	−0.17 to 0.08	0.499	–	–	–

^*^With no as the reference level. Bold values indicate significant *p*-values.

### 3.5 The effect of coaching combined with intervention effects

In order to explore the interdependency of the effects of coaching and interventions, both variables were included in bivariable linear mixed effects models. Results showed that the effect size of adjusting vaccination strategy hardly changed (−0.10 vs. −0.12) after adjustment for coaching, while the effect of coaching clearly became smaller and non-significant (−0.05 vs. −0.01; Table 4, model 1). In the bivariable model with internal biosecurity (Table 4, model 2) the effect of coaching reduced slightly (−0.07 vs. −0.05) and the association remained negative, while the effect of biosecurity hardly changed (−0.27 vs. −0.29) and remained statistically significant.

**Table 4:** The effect of coaching in combination of individual interventions, bivariable analysis adjusted for animal category.

**Model 1**				**Model 2**			
	**Estimated coëfficiënt**	**95% CI**	***p*** **-value**		**Estimated coëfficiënt**	**95% CI**	***p*** **-value**
Adjusting vaccination strategy	−0.12	−0.23 to 0.78	**0.033**	Biosecurity internal	−0.29	−0.54 to −0.05	**0.018**
Coaching	−0.01	−0.10 to 0.07	0.745	Coaching	−0.05	−0.12 to −0.01	0.110

Bold values indicate significant *p*-values.

## 4 Discussion

In this study coaching of a farm management team was used as a tool for the implementation of interventions to reduce AMU in farms with relatively high use of antimicrobials. Both coaching and interventions had an effect on AMU. After mutual adjustment, the associations between the individual interventions vaccination and internal biosecurity and AMU remained significant, while the effect of coaching decreased and became non-significant. These association patterns partially indicate that coaching did not have a direct effect, but most likely led to the introduction of interventions and these interventions did have an effect on AMU.

Coaches were used to intervene on behavior, by using a systematic plan for behavior change and stimulate the farm management teams to produce action plans and implement measures to reduce AMU. As a result, the coaching period was characterized by the introduction of a wide range of intervention measures, several of which are known to reduce AMU. We showed that coaching and the resulting interventions explained the effect on reducing AMU to a large extent compared to the effect of coaching itself. Multiple interventions were univariably associated with a reduction of AMU in this study, i.e., changes in feed, adjusting vaccination strategy, treatments, internal biosecurity and development of management team. However, in the multivariable model, the significant effect on AMU was only seen for adjusting vaccination strategy and internal biosecurity. This does not exclude the fact that other interventions played a role in reducing AMU, the limited sample size of the study might have been of influence. Adjusting vaccination strategy and internal biosecurity have been identified as important interventions for AMU reduction, which is consistent with a recent study by Dewulf et al. (29). In this study, 116 pig health experts from different countries were asked to rank alternatives for antimicrobial use in pig farming. The highest ranked alternative was biosecurity followed by an improved vaccination plan. However, the success of an intervention may depend on the amount of gain which can still be made on that specific intervention. For instance, when the internal biosecurity is already optimized in the farm, intervening in that specific management item will not have a big effect. In the future, more detailed research is necessary on the tailor-made approach whereby both the possible role of a coach and the effect on implementing management changes is being examined on their separate contributions to antimicrobial reduction.

AMU in pig farming has been decreased substantially each year since the start of sector-wide AMU monitoring in 2009. Although the decrease was highest at the beginning, during the study period AMU still decreased nationally on a yearly basis (9, 22, 26, 27). However, the effect of time on AMU was small during the non-coaching period compared to during the coaching period, indicating that these farms, had a relatively stable AMU pattern before coaching took place. Moreover, when analyzing the effect of time and the effect of coaching bivariable, the effect of coaching remained and the effect of time disappeared. These results suggest that the decrease was driven by coaching and not time itself.

Within Europe, different approaches have been described to reduce AMU in the last decade. Those studies were mainly performed in a time period when the group of farms with high AMU was large, quick wins on interventions for farmers were easily made, and the majority of the farmers were able to make changes on reducing their antimicrobial use (30–32). As seen in the last few years in the Netherlands, the proportion of high user farms declined and a specific group of structurally high users remained (9, 22, 26). For a small group of farmers with fluctuating and high AMU, a tailor-made approach might be more efficient in reducing AMU. In this study we made an initial attempt to combine the behavioral side of working in a management team and a tailored and structured animal health plan for each farm. This unique combination led to the implementation of interventions which had positive impact on reducing AMU.

Participation to this study was on a voluntary basis. A lot of effort has been made to recruit and incorporate eventually 57 farms. Farmers were reluctant to participate due to lack of interest, lack of implications for being a high user at the start of our study or due to other for the farmer more important issues that demanded attention and farmers that were not willing to invest the time. Because of the size of the group of participating farms, a stepped wedge design was applied as used earlier with success in companion animals (23). It is known that stepped wedge intervention studies are more powerful than a parallel design (with an intervention and control arm) when clusters are less homogeneous (33).

Farmers were recruited by their veterinarian or enlisted themselves after contact with the project members, the farmers thereby might have been more interested and motivated to participate and were willingly to implement the proposed interventions. If the approach was imposed to farmers by legislation or otherwise, the willingness to implement interventions might be different with less results in AMU. In this study, very high users of antimicrobials, users with a marginally higher AMU relative to the existing benchmark criteria (22), and users that were fluctuating between high and average AMU were recruited. This may have influenced the outcomes of this study. Especially when farms had a low usage at the start of coaching, the overall effect of intervening in a high AMU using farm might be underestimated. Besides, our study was performed during a period with several critical issues for the agricultural sector in the Netherlands, e.g., sharpening of nitrogen emission regulations, climate change related emission targets, and sell-out arrangements. This could have negatively influenced individual farms on the focus and progress of the study. The intervention period occurred simultaneously with the COVID pandemic, and resulted in online coaching sessions which might have had effect on the outcomes of the study as well. Follow-up time is likely too short to assess the sustainability of the effect of coaching.

## 5 Conclusion

Targeting high users of AMU via a voluntary route with a tailor-made approach reduced AMU. Adding coaches to the farms as done in this study added a more structured working method in the management team for implementing interventions. This study shows that coaching and related interventions were associated with AMU, although multiple factors may have played a role in the decrease.

## Data availability statement

The datasets presented in this study can be found in online repositories. The names of the repository/repositories and accession number(s) can be found at: https://doi.org/10.24416/UU01-RF2V6X.

## Ethics statement

The study was conducted in accordance with the Dutch law and institutional requirements. This had implication for data processing because of privacy issues and the study complied with the existing regulations. The studies were conducted in accordance with the local legislation and institutional requirements. The participants provided their written informed consent to participate in this study.

## Author contributions

HP: Investigation, Writing – original draft, Writing – review & editing, Conceptualization, Funding acquisition, Methodology, Project administration, Visualization, Data curation, Resources. HD: Writing – original draft, Writing – review & editing, Formal analysis, Data curation, Software. DH: Writing – review & editing, Funding acquisition, Methodology, Supervision. JW: Writing – review & editing, Funding acquisition, Supervision. DS: Writing – review & editing, Supervision. WD: Writing – review & editing, Data curation, Methodology, Supervision.
