# Supplementary material for: A coaching approach to strengthen farm management teams to reduce antimicrobial use in Dutch high usage pig farms: a 2 year intervention study
Source: Front Vet Sci. 2024 Jul 29;11:1422756. doi: 10.3389/fvets.2024.1422756 (PMC11317431; doi:10.3389/fvets.2024.1422756)
Supplement: Supplementary file 1 [file Table_1.DOCX]

Table 1 Overview of the 13 interventions groups with short description

| Variable | Explanation |
| --- | --- |
| Making checklist/protocol | Introducing and implementing checklist or protocols |
| Biosecurity check | Validated risk-based biosecurity quantification tool “Biocheck.UGent^tm^” |
| Change in feed | Changes in feed or drinking composition safety or quality |
| Monitor animal data | Monitoring technical animal performance data and discussing it in the management team |
| Cleaning | Cleaning and disinfection of the feeding utilities, disinfection methods |
| Adjusting vaccination strategy | Critical reflection on vaccination strategy and adjusting vaccination strategy |
| Treatment | Improved health treatments and diagnostics |
| Contact structure | Age and transfer management of the (young) animals |
| Biosecurity external | Biosecurity extern, loading and unloading animals, transport, pest control |
| Biosecurity internal | Biosecurity intern, separate compartments, shower facilities employees |
| Climate | Improved climate and environmental conditions |
| Development management  team | Development of management team on process and collaboration |
| Change management | Change of management team, change of persons (veterinarian, feed advisor) |
